# How Economic Analysis Can Contribute to Understanding the Links between Housing and Health

**DOI:** 10.3390/ijerph14090996

**Published:** 2017-08-31

**Authors:** Ralph Chapman, Nicholas Preval, Philippa Howden-Chapman

**Affiliations:** 1 Environmental Studies Programme, School of Geography, Environment and Earth Sciences, Victoria University of Wellington, Wellington 6140, New Zealand; 2 Department of Public Health, University of Otago, Wellington 6242, New Zealand; nicholas.preval@otago.ac.nz (N.P.); philippa.howden-chapman@otago.ac.nz (P.H.-C.)

**Keywords:** housing, health, cobenefit, cost benefit analysis, discounting, carbon, mental health, value of life

## Abstract

An economic analysis of housing’s linkages to health can assist policy makers and researchers to make better decisions about which housing interventions and policies are the most cost-beneficial. The challenge is to include cobenefits. The adoption in 2015 of the UN Sustainable Development Goals underscores the importance of understanding how policies interact, and the merit of comprehensively evaluating cobenefits. We explain our approach to the empirical assessment of such cobenefits in the housing and health context, and consider lessons from empirical economic appraisals of the impact of housing on health outcomes. Critical assumptions relating to cobenefits are explicitly examined. A key finding is that when wider policy outcome measures are included, such as mental health impacts and carbon emission reductions, it is important that effects of assumptions on outcomes are considered. Another is that differing values underlie appraisal, for example, the weight given to future generations through the discount rate. Cost-benefit analyses (CBAs) can better facilitate meaningful debate when they are based on explicit assumptions about values. In short, the insights drawn from an economic framework for housing-and-health studies are valuable, but nonetheless contingent. Given that housing interventions typically have both health and other cobenefits, and incorporate social value judgements, it is important to take a broad view but be explicit about how such interventions are assessed.

## 1. Introduction

For better or worse, economics so far remains dominant as the core language of policy making [1], perhaps because economic efficiency and growth are valued so highly in most political contexts. In particular, cost-benefit analysis (CBA) provides a way for governments to reach “rational” decisions on allocation of scare resources across a number of areas using a common language of benefits and costs, valued in dollar terms. CBA is focused on the estimation of “net welfare benefits”, an approximate indication of a policy’s estimated benefits to society as a whole, after accounting for the resource costs to society. Ministers, particularly Ministers of Finance, need to compare and triage government budget proposals from a wide range of agencies. Both investment and regulatory proposals can be analysed within a CBA framework, prioritising those with the greatest likely net benefits and excluding those likely to yield low net benefits. In short, CBA can provide powerful evidence for policy adoption (or otherwise) and is advocated in the housing domain by organisations such as the Organization for Economic Co-operation and Development (OECD) [2]. 

Thinking in terms of net welfare benefits to society represents an advance over traditional narrowly focused government thinking, for example where government ministers ignore cost impacts beyond the remit of their office or the government sector or wider social benefits. Such thinking has often been unjustifiably narrow. The full social benefits of expenditure should be estimated if at all possible [3]. CBA enables governments to appraise, using the same dollar units, the welfare cobenefits and costs associated with investment in a sector such as housing (be they benefits for health, wellbeing, energy, emission reduction, gains to employers in terms of reduced absenteeism, or other gains) and compare these with the benefits and costs from investment in other sectors, such as transport. Not all cobenefits will be significant, but many are, and ignoring them may badly misrepresent the merits of a given policy from a societal perspective [4,5]. 

CBA has been powerful for ministers and other policy makers because it can counteract the special interest pleading which has traditionally dominated policy making and still does in many jurisdictions [6]. CBA is also powerful because, in principle, it enables a government to think systematically about future benefits and costs, and compare them with current benefits and costs. CBA has a consistent way of valuing costs and benefits that occur in the future—i.e., by “discounting”. Future values are reduced, using a discount factor, to today’s terms, thereby accounting for people’s preference for consumption today rather than in the future. 

One of the advantages of CBA is that it is a framework that is largely independent of scale. For example, an investment may be small, but have a high benefit to cost ratio (BCR) and may be worth doing even though the absolute magnitude of benefits may be limited. Public health, according to Geoffrey Rose, should address policies that have an impact on the population as a whole on the basis that addressing the cause of individual illness is not the most effective way of improving the health of the whole population [7]. This means CBA tends to be consistent with the public health approach, as CBA aggregates benefits across the whole population. In principle, it supports taking into account the economies of scale to be gained by addressing the structural determinants of health and wellbeing across a large number of people. At the same time, just because CBA suggests that local policies may create scalable health benefits, it should not be forgotten that additional benefits may accrue from national level coordination to systematise policy design and support take-up.

In practice, the conceptual coherence of CBA can be undermined by a shortfall of empirical evidence about cobenefits [8] (p. 6), various practices such as downplaying (or cherry-picking) social and environmental costs and benefits, and by various assumptions. One assumption that we do not examine further here is that one person’s utility gain can compensate for another’s loss: this is a crude simplification, which has been widely criticised for its distributional insensitivity [9]. Another assumption, which this paper discusses further, matters considerably in practice in regard to long-term policy appraisal—the use of high discount rates. Discount rates are sometimes set too high when what are in fact social choices are construed as individual or market choices. For example, the decision to curb or not curb climate change is a critical social choice and will undeniably have irreversible and life or death consequences for many people in decades and centuries to come. Discounting at a high rate in appraising climate-relevant policies downplays such future social harms. It assumes that a market-based interest (or discount) rate is relevant to a social choice. However, as many have argued, there is no ethical basis for valuing a future life significantly less than one lived today. (For example, at a discount rate of 5 percent per year, the death of over 2 million people 300 years from now becomes less serious than the death of one individual today) [10]. Social discount rates should therefore generally be lower than individual market discount rates. Even if human life is not involved, and rather more mundane social benefits are at issue, discount rate overestimation is a problem with regard to many interventions with such benefits( for example, policy measures to support retrofitting insulation in houses to make them more energy efficient and health-supporting). As well as improving individual health (e.g., reducing mortality) over time, such interventions can contribute to a healthier population and reduce carbon emissions, i.e., generate beneficial future impacts of a social nature. If such future effects are not sufficiently weighted, policy decisions may be based on an underestimate of the net social benefits of the intervention [11]. Coherence in assessing future effects of social policy measures is essential for housing and health, because housing is a long-life asset that generates impacts on health as well as other outcomes—such as energy usage—over time frames running over decades.

Recently, there has been increasing attention paid to cobenefits in contexts such as social return on investment (SROI) analysis [12,13] (despite few analyses as yet in the housing domain); and in the formulation and analysis of the UN Sustainable Development Goals (SDGs). Globally, there is increasing awareness of how social, ecological and technological systems are best seen as interconnected, in their resourcing, their impacts and their evolution [14,15]. Despite this, there is a risk that the sustainable development goals will be viewed as a series of distinct goals to be addressed separately by different parts of government and perhaps different business sectors and non-governmental organisations (NGOs). However, interactions between SDGs are critical, and the 17 goals need to be seen as an integrated set. For example, from a public health perspective, SDG 3 (health) has strong interdependencies with SDG 11 (resilient and sustainable cities, which encompasses housing) [16].

The main aim of this paper is to use case studies to examine in more depth and more concretely, in the housing and health context, questions about assumptions and their implications. Accordingly, we describe and discuss the approach we have adopted in selected community housing studies and draw conclusions about the methodological issues in undertaking CBAs of housing interventions with multiple cobenefits. These housing-related studies were undertaken by He Kainga Oranga/Housing and Health Research Programme, a research group centred at the University of Otago, New Zealand, together with collaborating researchers. We are conscious that with sound CBA evidence, policy interventions can have a rapid take-up by policy makers and that policy proposals without CBA evidence were unlikely to be as persuasive. The weight given to CBA, in a context where policy makers may sometimes lack appropriate evaluation skills, meant that we emphasised analysis based on high quality evidence gathered from community trials and evaluations, together with clearly signposted, conservative assumptions. 

## 2. Methods

For this paper, we selected two main case studies to examine and discuss the issues around evaluations of housing interventions with multiple cobenefits. The case studies were selected to illustrate the significance for health and sustainability outcomes of key assumptions made in the evaluations. The case studies themselves were two of a number of community trials carried out by He Kainga Oranga. One was an appraisal of a government energy efficiency programme that was in place on a discretionary basis but had not been rolled out nationally. Our study of this intervention, the “Housing, Insulation, and Health Study” [17,18,19], was an ex ante appraisal of whether the intervention would be cost-beneficial if the cobenefits of the programme were taken into account. The second case study was a quasi-experimental *ex post* evaluation of the national roll-out of the “Warm Up New Zealand” (WUNZ) programme, which built on both our “Housing Insulation and Health Study” and the later “Housing, Heating and Health” community trial [20]. In each case, we carried out CBAs [19,21,22]. The measures in these CBAs are examined closely here with the aim of illuminating the central research question: what are the merits and limitations of CBAs of housing interventions, especially with regard to treatment of cobenefits, and how are reported outcomes influenced by assumptions in regard to the values of key parameters such as the discount rate and the value of human life? We selected the case studies because, across the range of studies undertaken by the authors of this paper, they represented those housing and health studies which most clearly illuminate the advantages and disadvantages of CBAs. 

Our general approach to conducting each of the CBAs was to estimate carefully the marginal benefit accruing to society (rather than the marginal private benefit) of a housing investment such as insulation. We took this approach to both the insulation community trial and the evaluation of the national roll-out of the housing insulation programme. We therefore compared the costs of interventions with the value of those reductions in hospitalization costs, general practitioner visits, days off work and school, pharmaceutical costs, energy costs, and even mortality, which could be ascribed to the housing investment in question. Evaluating some of these variables required independent measures as well as self-reports in some cases. Electricity and gas company records were requested to gain metered energy data for the insulation study. Schools were visited to independently gather administrative data of children’s days off school [23]. To facilitate this process, economists who contributed to study designs were included in the multidisciplinary research teams [24]. The choice of outcome variables was based on a broad “determinants of health and well-being” framework that incorporated economic outcomes alongside social ones [25].

Many of the cobenefits tend to be overlooked in simpler analyses as they benefit society as a whole or, in the case of avoided mortality, are very difficult for the individual to appraise and are thus easily overlooked. An analytical perspective emphasizing social benefits is more policy relevant in countries such as New Zealand, the UK, and northern Europe, where the state supports health care. In other countries, such as the USA, which use an insurance model for covering health care, the focus is on private benefits, so that social benefits are more likely to be downplayed or discounted.

An underlying assumption was that, in the absence of the investment, a quantity of insulation or an effective heater would be supplied in the private housing market (at a marginal private benefit above or equal to the cost of the intervention). However, because of the social value of additional insulation or heating, in terms of the health and other benefits just noted, a greater quantity of insulation or heating may be warranted at the “economic optimum” and, if a study finds that a policy action generates a net benefit (a benefit cost ratio significantly greater than one), it should in principle be considered for subsidy by the state in order to maximize benefit to society as a whole.

Key issues of interest relate to the interpretation of suggestive results, the consideration and valuation of carbon emission reductions, the effect of the discount rate in the treatment of benefits and costs that accrue in the future, and the valuation of human life. We introduce these issues here and draw conclusions about them using the case studies in the Results section.

One of the limitations of basing policy on community trials is that there are dangers in relying on studies not statistically powered to detect some outcomes. For example, health impacts such as hospitalization is a relatively uncommon event among any group of people, and community trials powered for more common health outcomes such as wheezing will not necessarily pick up reductions in hospitalization at a statistically significant level, even if the trend is in that direction. Often, studies with many thousand participants will be needed to detect such a hospitalization effect. Such studies are costly to undertake, and few are undertaken (the evaluation of the WUNZ programme presented below is an exception); consequently, there may be an erroneous presumption that such impacts are non-existent or too small to be taken into account. In other words, there is unlikely to be adequate investment into research studies that can be used to illuminate critical outcomes that can greatly influence the results of CBA, and the erroneous presumption tends to be that, in the absence of any statistically significant demonstrated effect of an intervention, there is no effect [26]. 

These considerations relate to the broader issue of what social outcomes are or are not “counted”, or even considered, in housing-related CBAs. In general, the principal limitation of such CBAs is that several less tangible, or intangible, effects of housing interventions are not able to be fully quantified and reliably valued, and are thus—in practice—often heavily downplayed. Health aspects are one, and others relate to energy use, illustrated below. There is of course a risk that interest groups could exaggerate beneficial impacts, but given the difficulties of establishing and conveying conclusive evidence about these impacts in the real world of limited research funding, and limited attention on the part of government officials, there is a case for giving considerable weight in policy assessment to suggestive (even if not conclusive) empirical results from a careful scientific study. 

To focus on carbon savings as an instance, it is increasingly important that such savings associated with energy use be considered carefully. Housing operation involves considerable energy use, and insulated housing and more efficient home heating can save energy. Three points are important. First, because energy savings from housing interventions may be modest at the individual household level, they are often seen as modest even across society at large. In the case of fossil fuel–based energy savings, our societal perspective has begun to change as carbon emission reductions have become more critical, with the emergence of climate change as an urgent environmental and social issue [27,28,29]. An accumulation of small emission reductions may add up to a useful contribution to emission reduction goals, especially given that most countries will struggle to achieve their targets under the Paris Agreement, and the sum of these targets so far falls short of what is necessary to meet the collective goal of holding warming to “well below 2 °C”, leaving aside the more ambitious aim of limiting the temperature increase to 1.5 °C [30].

Second, the economic value of such contributions is increasing as prices of carbon and estimates of the social cost of carbon rise internationally. The social cost of carbon is an estimate of the (global) damage caused by a marginal increase in carbon emissions, and such an estimate allows us to place a value on policy actions reducing carbon emissions. Between 2010 and 2013, the U.S. government’s Interagency working group increased its estimate of the social cost of carbon by around 60%, to reflect improved “integrated assessment modelling” [31]. The new middle range values are around US$54/tCO_2_ in 2007 dollars by 2020 (or NZ$88/tCO_2_ in 2017 dollars) and should double by 2050. Even at these revised higher levels, it is likely that the numbers represent a conservative assessment of the true social cost of carbon, as has been widely noted [32,33,34].

Third, some economists emphasize that a large influence on the perceived social cost of carbon is ethical judgment about how society should value the interests of future generations [35] and note that the outcomes of CBAs are highly sensitive to parameter values—such as the choice of discount rate or equity weightings across different social groups—which can be considered an ethical matter. Disagreements about the values of such parameters can be addressed by using sensitivity analysis, which reveals the implications of different parameter values without taking a fixed position on them.

## 3. Results

Our case studies examine these points in more depth and more concretely. First, we discuss the cobenefits of carbon emission reductions associated with the appraisal of housing insulation, and the factors influencing the size of this cobenefit. Second, we consider outcomes that rely on self-report that cannot be independently measured. Third, we discuss the uncertainties and debates over attaching a value to human life, and the methodology for calculating the value of a statistical life (VOSL), where the principle of valuation is accepted, and the importance of equity-weighted evaluations.

### 3.1. Assessing Carbon Emissions in Housing-Related Community Trials

Carbon savings from more sustainable insulation and heating arrangements in residential and commercial buildings can contribute to the urgent process of reducing global carbon emissions. For any given intervention the impact will be limited, but worldwide, the contribution could well be significant [36]. At the household level, carbon emission reduction is unlikely to represent a significant driver of investment choices or behaviour change [37]. But it is important that a CBA of a policy influencing household level action does not neglect climate change mitigation as a cobenefit. Increasingly, public health experts are pointing to the enormous implications of climate change for long term public health [8,38,39]. 

Our analyses set out first to evaluate the change in energy consumed by households. In the Housing, Insulation, and Health study [19], we found that savings in metered energy (mains gas and electricity) were significant (of the order of 13%). Carbon savings benefits were estimated based on the fossil fuel savings (in non-renewable electricity and mains gas). Valuation assumed a value of NZ$30 per tonne of CO_2_, in 2002 dollars and, even converted to NZ$41 per tonne in 2017 dollars, this is a conservative estimate in relation to the relevant social cost of carbon estimates over the 30-year horizon of the assessment (insulation is estimated to last at least this long, and the social cost of carbon is now likely to be at least twice the NZ$41 level, as noted above). The discounted present value of the benefits of carbon savings were estimated at around NZ$100 (US$73) per household in 2002 terms, modest but not trivial compared with the net benefits (gross benefit less cost) of the intervention of NZ$1574. At today’s social cost of carbon, they would be over NZ$200. Reductions in peak period electricity demand generate electricity system benefits (savings for peak generation capacity and the distribution system), a regional public good, but these were too difficult to quantify. The overall benefit cost ratio (BCR) for those outcomes of the intervention which could be quantified was almost 1.87:1 at the time, and this ratio would have risen with the higher value of the social cost of carbon. Table 1 indicates a BCR of about 1.94 at the higher social cost of carbon (case C).

Table 1 also illustrates how the choice of discount rate matters. While the “base case” used a 5% discount rate, it can be argued that 3.5% is not unreasonable given that society’s discount rate will be lower than market interest rates. The UK Treasury’s recommended discount rate for social investment is also 3.5% [40]. Using this discount rate lifts the BCR estimate from 1.87 in the base case to 2.24.

### 3.2. Measuring Self-Reported Outcomes

One of the key decisions when undertaking a CBA is whether to include self-reported measures. Subjective health and well-being and self-reported mental health are of increasing interest to a range of policy advisers, including economists, partly because there is increasing confidence in such measures [41,42]. The principal outcome on which the power calculation of our Housing, Insulation and Health community trial was based was the improved mental health and well-being that the intervention was hypothesised to provide, as a result of the increased warmth of the indoor environment [17]. We assessed the occupants’ mental health through a widely used measure of pre-clinical mental health and well-being, the mental health scale from the Short Form (36) Health Survey (SF36), which has been shown internationally to be valid and reliable [43]. We found a significantly higher mental health score among the intervention group, who had received retrofitted insulation in their homes, than among the control group [17]. However, while we included independent measures of indoor temperature, we did not include the SF36 measure in our CBA, on the basis that as the study participants were not blinded as to whether they were in the intervention or the control group, this could have differentially affected their mental health. We made the conservative decision to rely on independent outcome measures, such as the amount of metered energy used and visits to the general practitioner. Even so, the benefit cost ratio of this trial was nearly 2:1. Largely on the basis of these results and the positive results of the subsequent Housing, Heating, and Health study, the programme was rolled out nationally. However, in the interim period the government agency that managed the insulation programme commissioned a CBA estimate from us that included the mental health outcomes [44]. 

This mental health analysis showed that the intervention group, who had insulation retrofitted in their houses, had 0.56 times the odds of being in the bottom half of a reduced mental health scale (0.41–0.77; *p* = 0.0003) [44]. This can be interpreted as a 44% reduction in the risk of poor mental health following the retrofit. In other words, while both groups had had similar mental health symptoms at the outset, there were only about half the odds of having such symptoms after insulation was retrofitted. This is broadly consistent with the results on the Role Emotional scale, where the scores before and after the intervention were also significantly different, on average by 11 percentage points (*p* < 0.0001). Consequently, we concluded that for those in uninsulated housing, the incidence of anxiety and depression is around twice that in the rest of the population. This is a highly significant result, given the rising levels of anxiety and depression in developed countries [45] where intervention has been found to be effective [46]. To exclude mental health/well-being as a possible variable in the insulation CBA simply because it is self-reported has the effect of underestimating the likely benefits of the intervention. In short, a positive value on the mental health benefits and a less conservative CBA could be justified. Table 1 illustrates how the benefit cost ratio (BCR) would increase significantly to 3.58 if estimated mental health benefits of insulation were included.

Note also that if the cobenefits for mental health and carbon savings are combined, together with a more appropriate and lower social discount rate of 3.5%, the overall effect (Case D) would be to increase the BCR substantially, to 4.36.

### 3.3. Valuing A Human Life

We were part of the team that carried out an ex post evaluation of the health impacts of the $343 million Warm Up New Zealand: Heat Smart (WUNZ) programme. This evaluation looked at the outcomes from insulating the first 45,000 houses in the programme (matched to over 216,000 uninsulated control houses) and enabled us to assess the impact of insulation and heating retrofits on rare events. In particular, given New Zealand’s pattern of excess winter morbidity, we were interested in testing whether insulation and/or heater retrofits funded by WUNZ would affect the mortality risk of vulnerable individuals. Using previous research by our group [47,48], we identified older New Zealanders with a pre-existing cardiovascular or respiratory condition as those most likely to benefit from the programme. When we compared the post-treatment mortality risk for those who received treatment with equivalent individuals in the control group we found that insulation retrofits appeared to reduce mortality risk for people aged 65 and older who had a pre-treatment circulatory related hospitalization. The hazard ratio (the ratio of the rate of death for treatment group individuals versus that of control group individuals) was 0.67:1 (*p* < 0.001), suggesting a strong protective effect. 

In order to incorporate this finding into the CBA that we had been commissioned along with others to produce, it was necessary to address both philosophical and practical questions regarding the valuing of mortality. We predicted that those individuals who avoided mortality might be expected to live approximately half the typical additional life years for people of their age: this meant an average gain of five years per person. We then had to ask how these five years might be valued. We adopted the most commonly used approach in the New Zealand context, using a figure known as “the value of a statistical life” (VOSL), typically used in the transport sector to capture the value of changes in mortality predicted to result from roading projects. New Zealand’s VOSL figures were calculated using the “willingness to pay” method, in which survey participants’ VOSL estimates were revealed using a series of questions [49]. We derived a value for a statistical life year (VOSLY) from this VOSL estimate and then applied the assumption just described, that each avoided death would result in a benefit of five VOSLYs. In taking this approach we deliberately avoided making any adjustment to VOSLY figures to account for the health of the individuals in question, as would be done when using other common measures such as quality or disability adjusted life years. VOSL literature is equivocal on this question, with some research suggesting an “inverse U” for VOSL when plotted against age [50], suggesting that older people value their lives less; however, this finding was not replicated in the New Zealand context [49,51]. Some research has found a link between age and VOSL, but not between chronic health condition status and VOSL [52]. In the light of this mixed evidence we chose an equity-oriented approach, assuming that the role of government was to distribute benefit to all members of the community equitably and that valuing all life years equally was the approach most consistent with this position.

Valuing reductions in mortality captured what proved to be the largest health benefit in a CBA of the WUNZ programme: in discounted net present value terms the programme gave a very favourable benefit cost ratio of close to 4:1 [53]. Indeed, further analysis suggests that, driven by mortality reductions for people over 65 years of age who had been hospitalised with pre-existing circulatory conditions in the previous year, the benefit cost ratio might be as high as 6.4:1 [54]. It is interesting to note that in the absence of a suitably large administrative dataset it would have been impossible to capture this benefit, leading to an incomplete CBA and quite possibly to an end of the WUNZ programme earlier than has been scheduled. It is useful to reflect on the inevitable use of “best guess” modelling techniques in such a context: our research group was careful to consistently signpost assumptions in order to facilitate discussion and ensure replicability, but policy makers were inevitably focused on the benefit–cost ratios calculated, and subtleties were likely lost in the process.

## 4. Discussion

Economic analyses are often sought by government and, when available, tend to be persuasive in policy debates, as government agencies and Ministers emphasize economic perspectives and take the view that CBAs are the most rigorous available way of considering the alternative uses of resources. The sway of CBA findings in policy debate means that researchers who carry out CBAs have a responsibility to produce quality work with all assumptions signposted and caveats noted clearly. Even so, these caveats often seem to be ignored and results discussed only in terms of the benefit cost ratio. Uncertainties and error bounds on CBA results are typically given little attention. 

A key point in design of a CBA is assessing the counterfactual. The study of the WUNZ programme looked at the state of insulation and heating of the housing of a broad segment of the population, but was careful to count only the social benefit of outcomes that would not have occurred in the absence of the insulation and heating programme in question [22]. That is, the study predicted the proportion of homes that would have purchased adequate insulation and heating in the absence of the programme and excluded benefits accruing to such homes from the assessment. Another design, used in the insulation trial [19] and heating trial [20,21], is to estimate the social and private benefits of an experimental intervention programme that was otherwise not available to a given group and compare the benefits against the resource costs (which in the study were covered by a government subsidy) to calculate a benefit cost ratio. The control group provided the counterfactual.

A deeper issue is the fact that the primacy of economic evaluation is not universally accepted. Observers from disciplines other than economics may have alternative perspectives on the use of an economic framework in which the economic value attached to outcomes is given primacy [1]. Other disciplines may emphasize intangible outcomes such as social equity, a sense of social purpose, identity, and the intrinsic values of nature—which are usually given short shrift in decision making.

This perspective is well articulated by Norwegian institutional economist Arild Vatn, who reasonably sees CBA as a particular “value articulating institution”. It can be compared with other means of articulating values, such as multi-criteria analysis (MCA) [55,56], in which a range of criteria are considered and weighted by the appraising group. Scores of policy options against the criteria, and weightings of the criteria, are then assigned by the individuals in the group, or agreed by the group. CBA and MCA can be seen as two equally valid approaches, or rule structures, which facilitate the ascribing of value.

Such different methods arise from differing “rationalities” or worldviews and turn on answers to questions such as who should help decide what is being appraised, what counts as evidence, and how are conclusions reached? CBA fits within neoclassical economics and is based on an individualist, “rational” model of human nature in which an individual’s values and preferences are seen as given and fixed, values are commensurable, all decisions are trade-offs, choice follows individual utility, and the market generally best resolves valuation issues. However, from a “social constructionist” perspective, preferences evolve socially, with norms and expectations derived from institutions and often from social deliberation about options for action. The deliberation “forum”—and there are various sorts possible—may be better able to resolve valuation issues than a technocratic process such as CBA. Vatn describes MCA and CBA rationalities as “We” and “I” based, corresponding to the citizen and the consumer [55]. Choices by government and/or society as to how government interventions are appraised can matter considerably for how societies function, and in Anglo-Saxon countries, an individualist rationality is typically privileged, as demonstrated in the predominance of CBA. 

A different but equally important point worth underlining is that it is helpful to see housing policy interventions within a larger systems perspective on society, the economy and the environment. To the extent that such a systems perspective is accepted, we see a gradually increasing sophistication in the assessment of policy interventions [15]. For example, rather than seeing housing policy options as focused only on optimizing household welfare for a given time and place, it is more helpful to view housing policies as part of a set of government choices regarding outcomes from the urban built and social environment, with both short and longer term consequences for such outcomes. Housing-linked policy choices, in areas such as transport, infrastructure provision, urban planning, and climate change policy, can have profound implications for the welfare of households over time [57,58]. This perspective is allied to the point underscored by the authors of a recent report on the UN’s Sustainable Development Goals, namely that interactions among the goals is critical to their implementation [59].

## 5. Conclusions

Economic analysis—typically using CBA—of housing’s linkages to health remains a useful policy tool to guide government and community investment, but care should be taken to conduct it in a way that as much as possible measures broad social and environmental cobenefits [60], and routinely clarifies the relationship between assumptions and results, through sensitivity analyses demonstrated in this paper. In the context of today’s efforts to consider interactions and cobenefits across the 17 United Nations sustainable development goals, a special effort to apply economic analysis in a comprehensive and inclusive way is warranted. CBAs can be particularly persuasive when based on randomized community trials designed to establish causal relationships in order to appraise likely benefits and costs of policies. The positive results of community trials discussed here are demonstrably even more robust when confirmed by a quasi-experimental ex post evaluation applied to a national policy roll-out. 

Yet the collection of evidence adequate for comprehensive CBAs is costly and elusive, and questions remain about the variables that are included in and excluded from CBAs. There has been a tendency toward narrow appraisals because research resources are scarce. While quantitative appraisals of tangible outcomes have generally been favoured over more intangible and qualitative outcome appraisals, the exclusion of more elusive variables such as mental health and well-being effects in favour of easily measured outcomes highlights measurement issues and the value questions implicit in the choice of outcomes. Even when wider policy outcome measures are included, such as the impact of energy efficiency policies on carbon emission reductions, the nature of assumptions highlights the differing values that inevitably underlie such appraisal tools. An example is the weight given to future generations through the discount rate. 

CBAs can best function when they are based on explicit assumptions that can facilitate meaningful debate. However, it is also important to remember that they represent only one of the various evaluative approaches and value sets and should not be unduly privileged given the multiplicity of values and rationalities in society.

## Figures and Tables

**Table 1:** How the benefit cost ratio varies depending on critical assumptions (sensitivity analysis of the insulation study).

Case	Base Case	Sensitivity Analysis Cases			
		Case A	Case B	Case C	Case D
Assumptions	5% discount rate	**3.5%**	5%	5%	**3.5%**
	No mental health benefits counted	No mental health benefits counted	**Mental health benefits included**	No mental health benefits counted	**Mental health benefits included**
	Low social cost of carbon	Low social cost of carbon	Low social cost of carbon	**Higher social cost of carbon**	**Higher social cost of carbon**
Benefit cost ratio (BCR)	1.87	2.24	3.58	1.94	4.36

Varied assumption shown in bold for cases A to D.
